# Supplementary material for: Changes in mGlu5 Receptor Signaling Are Associated with Associative Learning and Memory Extinction in Mice
Source: Life (Basel). 2022 Mar 21;12(3):463. doi: 10.3390/life12030463 (PMC8955168; doi:10.3390/life12030463)

Dorsal hippocampus

mGlu5

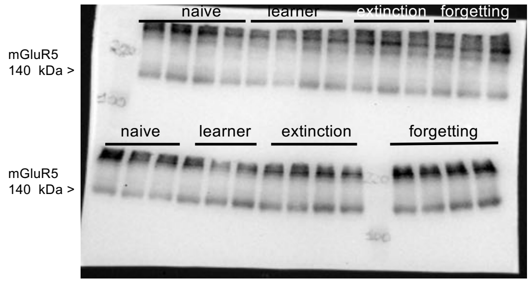

β-actin

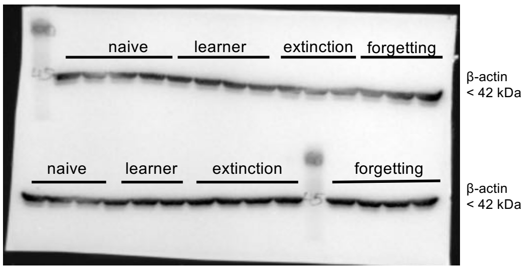

Norbin

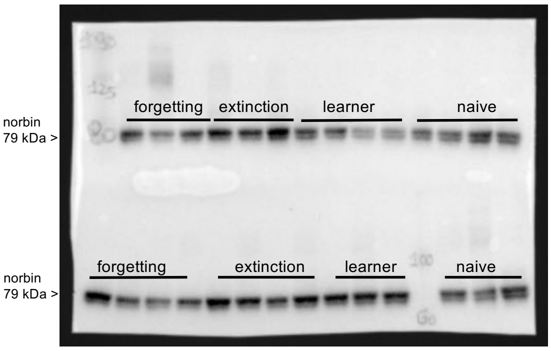

β-actin

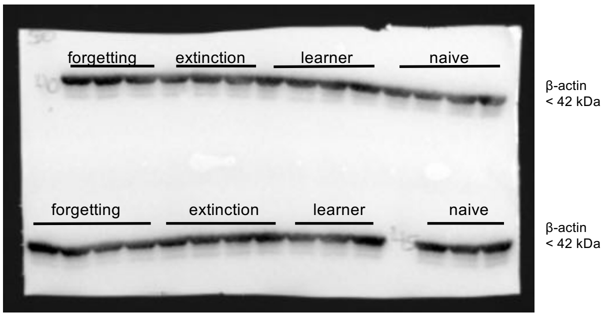

PLC-β

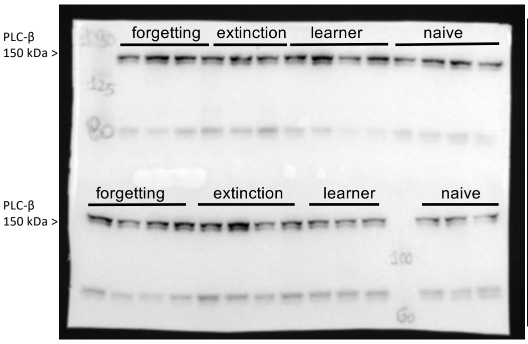

β-actin

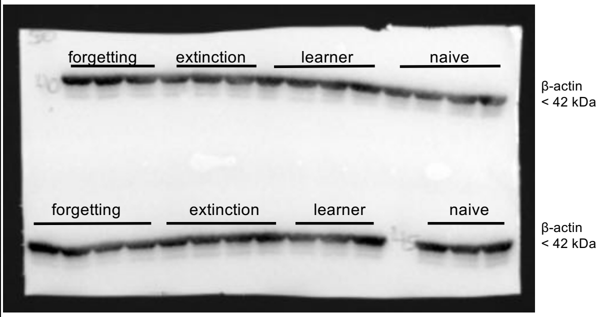

Gαq

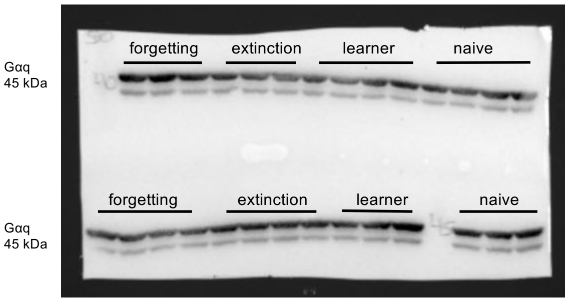

β-actin

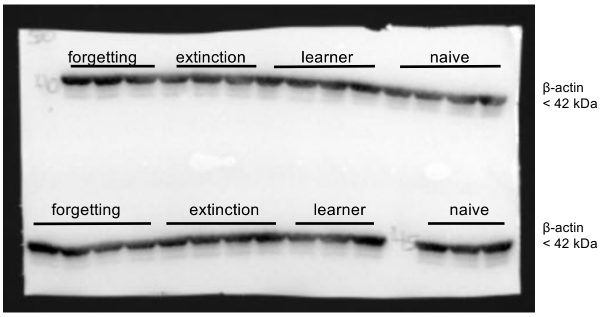

Ventral hippocampus

mGlu5

mGluR5
140 kDa >

mGluR5
140 kDa >

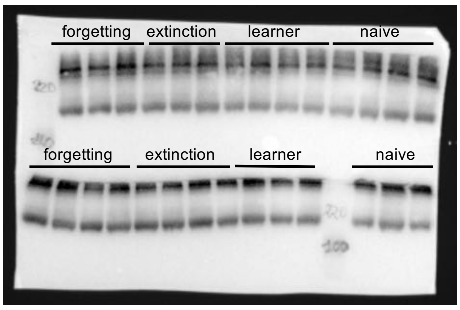

β-actin

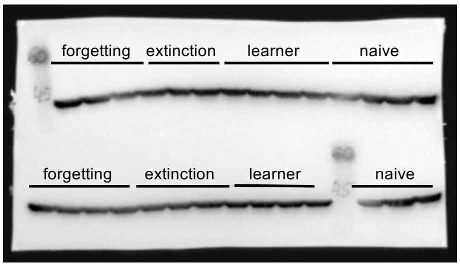

β-actin
< 42 kDa

β-actin
< 42 kDa

Norbin

norbin
79 kDa >

norbin
79 kDa >

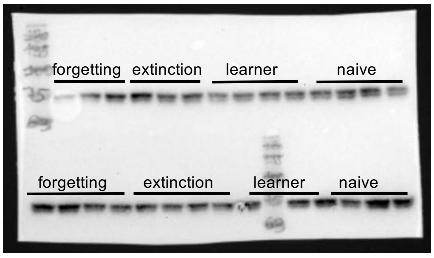

β-actin

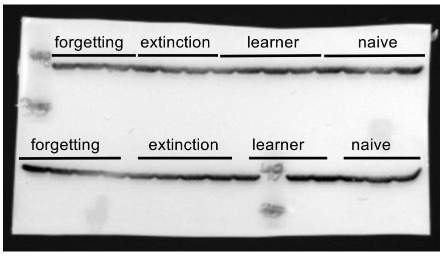

β-actin
< 42 kDa

β-actin
< 42 kDa

PLC-β

PLC-β
150 kDa >

PLC-β
150 kDa >

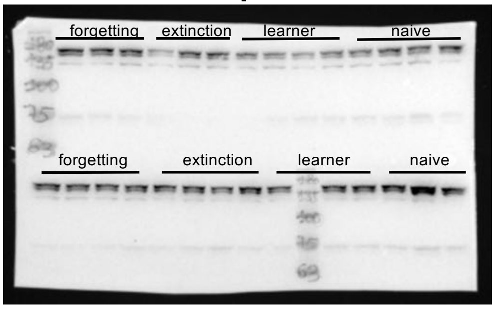

β-actin

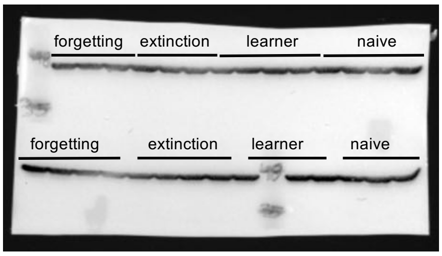

β-actin
< 42 kDa

β-actin
< 42 kDa

Gαq

Gαq
45 kDa

Gαq
45 kDa

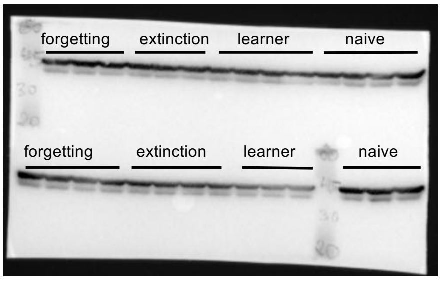

β-actin

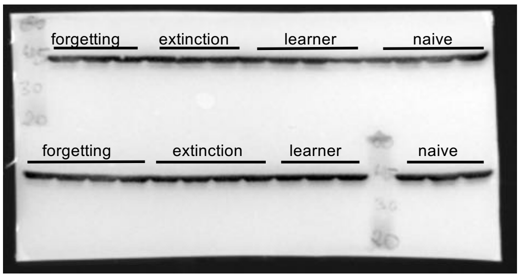

β-actin
< 42 kDa

β-actin
< 42 kDa

Prefrontal cortex

mGlu5

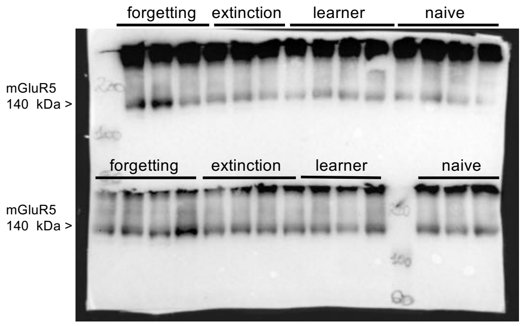

β-actin

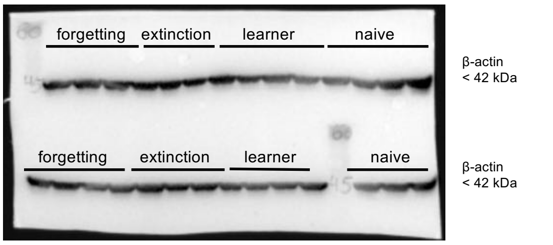

Norbin

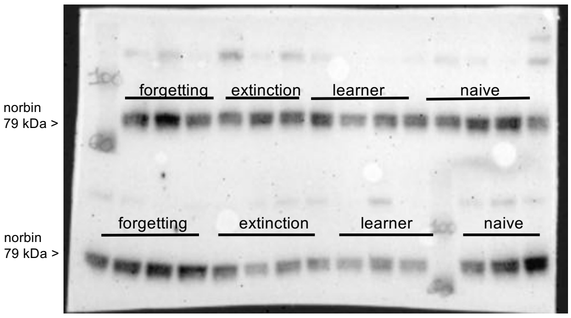

β-actin

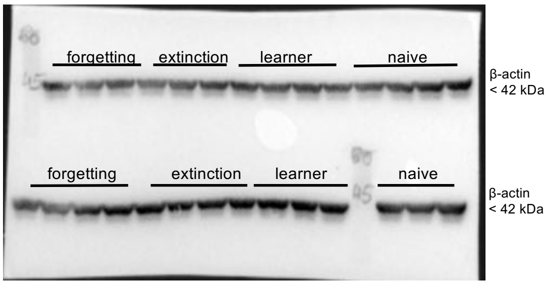

PLC-β

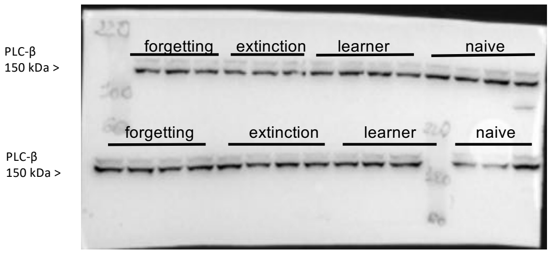

β-actin

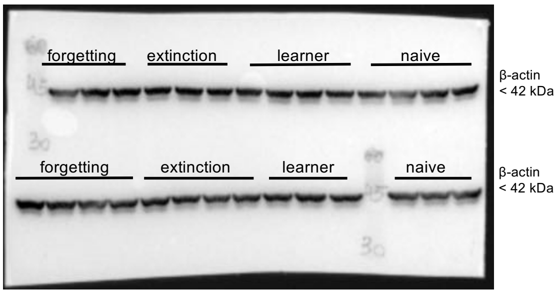

Gαq

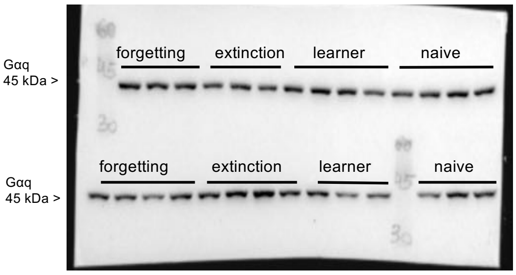

β-actin

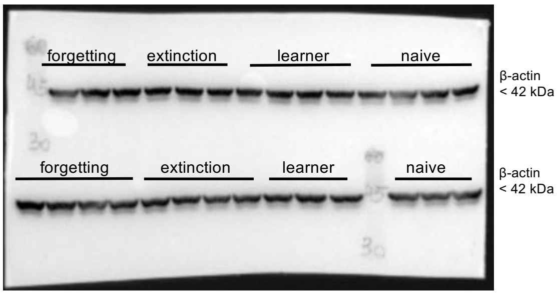

Supplement: Supplementary file 1 [file life-12-00463-s001.zip › Figure S1. Uncropped images of blots in prefrontal cortex, dorsal and ventral hippocampus.pdf]
